# Minimal/Measurable Residual Disease Monitoring in *NPM1*-Mutated Acute Myeloid Leukemia: A Clinical Viewpoint and Perspectives

**DOI:** 10.3390/ijms19113492

**Published:** 2018-11-06

**Authors:** Fabio Forghieri, Patrizia Comoli, Roberto Marasca, Leonardo Potenza, Mario Luppi

**Affiliations:** 1 Department of Medical and Surgical Sciences, Section of Hematology, University of Modena and Reggio Emilia, Azienda Ospedaliero-Universitaria di Modena, Via del Pozzo 71, 41124 Modena, Italy; roberto.marasca@unimore.it (R.M.); leonardo.potenza@unimore.it (L.P.); mario.luppi@unimore.it (M.L.); 2 Pediatric Hematology/Oncology Unit, Istituto di Ricovero e Cura a Carattere Scientifico (IRCCS) Policlinico San Matteo, Viale Golgi 19, 27100 Pavia, Italy; pcomoli@smatteo.pv.it; 3 Cell Factory, Istituto di Ricovero e Cura a Carattere Scientifico (IRCCS) Policlinico San Matteo, Viale Golgi 19, 27100 Pavia, Italy

**Keywords:** *NPM1*-mutated acute myeloid leukemia, molecular minimal/measurable residual disease monitoring, prognostic thresholds and timepoints, intensive chemotherapy, allogeneic hematopoietic stem cell transplantation, clinical outcome

## Abstract

Acute myeloid leukemia (AML) with *NPM1* gene mutations is currently recognized as a distinct entity, due to its unique biological and clinical features. We summarize here the results of published studies investigating the clinical application of minimal/measurable residual disease (MRD) in patients with *NPM1*-mutated AML, receiving either intensive chemotherapy or hematopoietic stem cell transplantation. Several clinical trials have so far demonstrated a significant independent prognostic impact of molecular MRD monitoring in *NPM1*-mutated AML and, accordingly, the Consensus Document from the European Leukemia Net MRD Working Party has recently recommended that *NPM1*-mutated AML patients have MRD assessment at informative clinical timepoints during treatment and follow-up. However, several controversies remain, mainly with regard to the most clinically significant timepoints and the MRD thresholds to be considered, but also with respect to the optimal source to be analyzed, namely bone marrow or peripheral blood samples, and the correlation of MRD with other known prognostic indicators. Moreover, we discuss potential advantages, as well as drawbacks, of newer molecular technologies such as digital droplet PCR and next-generation sequencing in comparison to conventional RQ-PCR to quantify *NPM1*-mutated MRD. In conclusion, further prospective clinical trials are warranted to standardize MRD monitoring strategies and to optimize MRD-guided therapeutic interventions in *NPM1*-mutated AML patients.

## 1. Introduction

The results of cytogenetic and molecular examinations at diagnosis of non-promyelocytic acute myeloid leukemia (AML), combined with the achievement of morphologic complete remission (CR) after remission induction chemotherapy, have significant prognostic impact on clinical outcome and usually serve to guide post-remission therapeutic strategies in younger adult patients [1,2,3]. However, the morphologic assessment of bone marrow (BM) blast percentage by light microscopy is significantly hampered by limited sensitivity and inter-observer variability, so that it is generally recognized that, when 5% myeloblasts is considered to be the cut-off to define morphologic CR, about 10^10^ leukemic cells may still persist in the patient [3,4]. The risk stratification in AML patients actually remains inadequate, and there is growing interest in the use of more sensitive laboratory tools, such as multiparametric flow cytometry (MFC) or molecular techniques, to detect low levels of residual disease in either BM or peripheral blood (PB) at different treatment timepoints [1]. The evaluation of minimal residual disease, also referred to as measurable residual disease (MRD), is considered useful to more precisely define AML response to intensive chemotherapy, thereby refining risk stratification [1]. Some studies have demonstrated that MRD persistence in a condition of morphologic CR confers high relapse risk and adverse prognosis, comparable to the one associated with persisting leukemic cells at microscopic examinations [4,5,6]. Interestingly, the recently revised European Leukemia Net (ELN) recommendations for AML management have introduced the category CR without MRD (CR_MRD−_) [7]. Moreover, long-term MRD monitoring in patients in CR may serve to early detect leukemia relapse [1]. MRD assessment captures the diversities of the underlying cytogenetic/genetic AML characteristics and also recapitulates patients’ heterogeneity regarding chemotherapy bioavailability, metabolism and resistance, thus resulting in a unique in vivo tool to evaluate chemosensitivity of leukemic cells [4,8,9]. Moreover, even within homogeneous genetic subgroups, the long-term outcome of AML patients depends on the clearance of the molecular lesion, as clearly demonstrated in other myeloid neoplasms, namely chronic myeloid leukemia and acute promyelocytic leukemia [1,4]. Beyond MFC, several molecular techniques are currently available for MRD determination in AML patients, including real-time quantitative polymerase chain reaction (RQ-PCR), which is highly sensitive, reliable, rapid and reproducible between different laboratories, as well as newer but less standardized tools such as next-generation sequencing (NGS) and digital droplet PCR (ddPCR) [2,4]. RQ-PCR allows MRD detection in patients with documentation of chimeric fusion genes generated by balanced chromosomal rearrangements, especially in cases of acute promyelocytic leukemia and core-binding factor (CBF) leukemias, but also in AML cases with other genetic alterations, such as insertions/duplications (e.g., *NPM1, FLT3-ITD, MLL-PTD*), point mutations (*CEBPA*, *IDH1/2*, *KIT*, *RAS*, *RUNX1*, *TP53*) or gene overexpression (*WT1*, *EVI1*, *ERG*) [1,2,4]. While MFC, despite its limitations, could enable MRD assessment in the vast majority of AML patients, a major drawback of RQ-PCR is its applicability only to those cases, accounting for approximately 50–60% of younger AML patients, who bear at least one molecular lesion, specific and stable over the treatment course, which could reliably be monitored using this molecular technique [2,4,10].

## 2. *NPM1* Mutations in AML: Biological and Clinical Features

Nucleophosmin (*NPM1*) gene encodes for a protein which physiologically shuttles between nucleus and cytoplasm, acting as a molecular chaperone to establish multiple protein-protein interactions [11]. NPM1 protein is normally involved in critical cell functions, such as control of ribosome formation and export, stabilization of the oncosuppressor p14^Arf^ protein in the nucleolus, and regulation of centrosome duplication [12,13,14]. *NPM1* gene mutations, occurring in approximately 30% of adult AML cases, and in 50–60% of AML cases with normal karyotype, represent one of the most frequent molecular lesions observed in AML [14,15]. Since the discovery of *NPM1* mutations in 2005 by Falini et al. [16], more than 55 different mutations, mainly occurring in the exon 12 of the gene, have been described, but three mutation types (A, B and D) account for 95% of all cases [8,14,17]. *NPM1* gene mutations result in structural changes of the C-terminus of NPM1 protein, with subsequent aberrant cytoplasmic delocalization, leading to a unique immunohistochemical pattern detectable on BM trephine biopsy [16,18]. This cytoplasmic accumulation of NPM1-mutated protein causes perturbations of multiple cellular pathways through a combination of loss of functions and gain of functions, critical for leukemogenesis [12,13,14,17]. Notably, it was recently reported that NPM1-mutated protein dislocated PU.1 into cytoplasm with it, whereas CEBPA and RUNX1, the master transcription factors that collaborate with PU.1 to activate granulo-monocytic lineage-fates, remained in the nucleus. However, without nuclear PU.1, their coregulator interactions were toggled from coactivators to corepressors, thus repressing >500 granulocyte and monocyte terminal differentiation genes [19].

As expected for founder genetic lesions, *NPM1* mutations are specific, being almost exclusively restricted to AML, usually de novo, and generally expressed in the whole leukemic population [13,14,20]. Notably, *NPM1*-mutated AML, showing distinctive genetic, pathologic, immunophenotypic and clinical features, has now been recognized as a full distinct entity among AML with recurrent genetic abnormalities in the 2016 revision of World Health Organization (WHO) classification of myeloid neoplasms and acute leukemia [21]. While the infrequent presence of coexisting chromosomal abnormalities, observed in only 15% of patients, does not appear to modify the prognostic effects of *NPM1* mutations [7,22,23], prognosis may be significantly influenced by accompanying molecular lesions, mainly *FLT3* and *DNMT3A* gene mutations, documented in about 40% and 50% of *NPM1*-mutated AML cases, respectively [7,15,17]. In detail, the better risk outcomes observed in *NPM1*-mutated AML patients were generally considered limited to cases without concurrent *FLT3*-ITD mutations [17,24]. Furthermore, the deleterious prognostic effects of *FLT3*-ITD have previously been found to be most clinically relevant when co-occurring with *NPM1* and *DNMT3A* mutations, as opposed with either mutation alone [25]. However, recent studies have suggested that patients with *NPM1* mutation and *FLT3*-ITD with a low (<0.5) allelic ratio have a similar favorable outcome as patients with *NPM1*-mutated AML without *FLT3*-ITD [26,27,28,29]. Thus, both of these latter groups are currently considered favorable according to the 2017 ELN risk stratification, in contrast to *NPM1*-mutated AML associated with *FLT3*-ITD with high allelic ratio, which is characterized by higher relapse rate and poorer overall survival (OS) [7].

## 3. ELN Recommendations for MRD Assessment

Based upon the above-mentioned biological and clinical characteristics, especially their homogeneous mutation pattern in AML patients, *NPM1* mutations may be considered an ideal leukemia-specific target for MRD detection [2]. Since the first application by Gorello et al. of sensitive and specific RQ-PCR assays as a reliable system to quantitatively assess *NPM1*-mutated gene copies [30], several studies have investigated the clinical implications of MRD monitoring in *NPM1*-mutated AML undergoing intensive therapeutic approaches (Table 1) [30,31,32,33,34,35,36,37,38,39,40,41,42,43,44,45,46,47,48,49,50,51,52,53,54,55,56,57,58,59,60,61,62,63,64,65,66,67,68,69,70,71,72,73].

The consensus document from the ELN MRD Working Party indicates that AML patients with *NPM1* mutations, such as patients with *RUNX1-RUNX1T1*, *CBFb-MYH11* or *PML-RARA* fusion transcripts, should have molecular MRD assessment at informative clinical timepoints. During the active treatment phase, MRD assessment for these molecular lesions is recommended at minimum at diagnosis, after 2 cycles of induction/consolidation chemotherapy, and at the end of treatment [74]. Furthermore, after the end of treatment, samples for MRD analyses should in general be collected every 3 months for 24 months. Thereafter, monitoring beyond 2 years of follow-up should be based on the relapse risk of the patient and decided individually [74]. Specifically regarding *NPM1*-mutated AML, with or without other concomitant mutations, monitoring of *NPM1* transcripts is recommended in BM and PB, if possible. If *NPM1*-mutated MRD remains negative in PB but positive in BM after the end of treatments, transcripts should be closely monitored every 4 weeks for at least 3 months, in order to evaluate any MRD increase. Conversely, if a rising MRD is not confirmed or MRD becomes undetectable, then MRD retesting may be regularly performed at 3-month intervals for at least the first 2 years after the end of treatment [74].

At first glance, the clinical management of *NPM1*-mutated AML patients according to these recommendations could appear clearly defined. However, several controversial issues remain, especially with regard to the most clinically significant timepoints and MRD thresholds to be considered, but also with respect to the best source to be analyzed, namely BM or PB samples, and correlation of MRD with other known prognostic indicators (Table 2) [8].

## 4. Prognostic MRD Thresholds and Relevant Timepoints for *NPM1* Mutations

As above mentioned, several clinical trials have so far validated the clinical relevance of molecular MRD monitoring in *NPM1*-mutated AML patients (Table 2), trying to identify prognostic MRD thresholds and most relevant timepoints for collecting either BM or PB samples [30,31,32,33,34,35,36,37,38,39,40,41,42,43,44,45,46,47,48,49,50,51,52,53,54,55,56,57,58,59,60,61,62,63,64,65,66,67,68,69,70,71,72,73]. Schnittger et al. retrospectively analyzed 1227 diagnostic and follow-up samples in 252 *NPM1*-mutated AML patients by RQ-PCR assays [35]. A total of 47 relapses were predictable due to *NPM1* mutation levels increase of at least 1 log or in 15 cases because of a less than 3-log reduction in *NPM1* mutation levels. High prognostic value for MRD monitoring was documented at four different intervals after therapy initiation (namely 18–60 days, 61–120 days, 121–365 days, >365 days), with levels of residual *NPM1* transcripts as the most relevant factor affecting event-free survival (EFS) in multivariate analysis, also in the subset of patients undergoing hematopoietic stem cell transplantation (HSCT). Moreover, MRD thresholds of 0.01% and 0.1% *NPM1*-mutated/*ABL1* obtained during first-line treatment and after HSCT or second-line therapy, respectively, discriminated between prognostic subgroups, but without influencing overall survival (OS) [35]. Another German-Austrian group evaluated MRD prognostic role in 245 intensively treated adults with *NPM1*-mutated AML [40]. Achievement of RQ-PCR negativity after double induction therapy identified patients with low 4-year cumulative incidence of relapse (CIR), namely 6.5% compared with 53%, for subjects with persisting MRD positivity. This also translated into significant differences in OS (90% versus 51%, respectively; *p* = 0.001). Furthermore, according to Kronke et al., *NPM1*-mutated transcript levels were an independent prognostic factor also when analyzed after completion of consolidation therapy (4-year OS 80% and 44% for MRD-negative and MRD-positive patients, respectively) and during follow-up, when serial MRD monitoring allowed early prediction of AML relapse in patients exceeding an arbitrary cut-off value of greater than 2% *NPM1*-mutated/*ABL1* [40]. Shayegi et al. subsequently investigated the prognostic impact of different *NPM1*-mutated MRD cut-off values, among 155 AML patients treated within intensive treatment protocols [44]. They found that MRD levels >1% after completion of conventional chemotherapy were associated with increased relapse risk and significantly worse survival outcomes, whereas for patients tested after having undergone HSCT, the best prognostic cut-off value for disease-free survival (DFS) and OS was 10% *NPM1*-mutated/*ABL1* [44]. Interestingly, Hubmann et al. monitored molecular MRD during aplasia, after induction and consolidation chemotherapy, and during follow-up in 158 patients with *NPM1*-mutated AML [47]. The assessment of MRD, with a cut-off ratio of 0.01 for absolute *NPM1*-mutated ratios and a 3-log relative reduction, after induction therapy resulted the most appropriate checkpoint in order to identify patients in CR at high risk of relapse. Indeed, the authors observed 2-year CIR 77.8% and 26.4% for MRD-positive and MRD-negative after induction, respectively. In this series, early molecular response thus seemed to be more prognostically relevant than persistent MRD positivity after consolidation treatments [47]. Subsequently, Ivey et al. reported serial prospective *NPM1* mutation MRD monitoring in 346 AML patients enrolled in NCRI AML17 trial [54]. The major finding of this large clinical study was that persistence of *NPM1*-mutated transcripts in PB samples exceeding a value of 0.01% *NPM1*-mutated/*ABL1* after the second chemotherapy cycle was associated with a greater risk of relapse at a 3-year follow-up than was the absence of such mutated transcripts (82% versus 30%, respectively; *p* < 0.001). Notably, MRD positivity after two treatment cycles was observed in 15% of patients and also translated into lower OS rates in these subjects compared with MRD-negative patients (24% versus 75%, respectively; *p* < 0.001). Additionally, the presence of MRD at this testing timepoint was the only independent prognostic factor for death in multivariate analysis and superseded other adverse baseline cytogenetic or molecular factors, such as *FLT3*-ITD or *DNMT3A* mutations [54]. More recently, Balsat et al. reported on MRD evaluation in 152 *NPM1*-mutated AML patients, showing that patients who did not achieve a 4-log reduction in *NPM1*-mutated transcripts in PB samples after induction therapy (45% of study cohort) had a higher 3-year CIR (65.8% versus 20.5%) and a shorter 3-year OS (40.8% versus 91.2%) compared with patients who obtained an adequate MRD reduction [57]. Interestingly, the outcome of patients with a positive PB or BM MRD but with >4-log reduction in PB MRD after induction treatment had a similar risk of relapse to subjects with both MRD negativity and >4-log reduction in PB MRD. Notably, in the subgroup of patients with *FLT3*-ITD, only age, white blood cell (WBC) count and <4-log reduction in PB *NPM1*-mutated MRD after induction, but not *FLT3*-ITD allelic ratio, were identified as prognostic factors. Among the 71 patients with non-favorable risk AML according to ELN risk stratification, DFS and OS were significantly improved by allogeneic HSCT only in those with a <4-log reduction in PB MRD, whereas this benefit was not documented in those with an MRD clearance >4-log, thereby suggesting the potential use of early MRD investigation in PB as a predictive factor for HSCT indication [57].

Collectively, the results of the studies presented above confirmed that RQ-PCR is a reliable molecular tool to assess MRD in most patients with *NPM1*-mutated AML [35,40,44,47,54,57]. The MRD monitoring, by either a kinetic measure [32,35,47,53,57,68] or the obtainment of an absolute threshold of *NPM1*-mutated transcripts [31,33,35,38,40,44,47,48,49,50,51,52,54,55,56,58,61,63,65,66,67,69], was overall predictive of relapse and survival outcomes (Table 2). However, due to differences in patients’ cohorts and the lack of standardization of *NPM1*-mutated RQ-PCR MRD assays and analytical methods, any comparison between different studies remains extremely difficult, and evidence-based conclusions on the best MRD thresholds and timepoints cannot be definitely drawn [8,44,75]. Interestingly, in the study by Balsat et al., a positive MRD determination in BM and concurrent negativity in PB samples was found in 24.6% of cases [57], in concordance with the 14.5% reported by Shayegi et al. in MRD samples collected at different timepoints [44]. Absolute differences in *NPM1*-mutated RQ-PCR sensitivity generally favor BM over PB on the order of 0.6–1.5 log_10_ [44,54,57,75], so that BM aspirate was initially considered to be the best source to investigate MRD [75]. Also, in the series by Ivey et al., serial monitoring of PB and BM paired samples confirmed that the BM analysis increased the rate of detection of MRD, affording a median 1–log_10_ increment in sensitivity [54]. However, the presence of *NPM1*-mutated transcripts, with a 0.01% absolute threshold to define positivity, in PB of patients after the second chemotherapy cycle was highly discriminatory and was the only significant prognostic factor [54]. It has thus been hypothesized that MRD assessment in BM could overestimate the quality of response due to the risk of BM samples dilution by PB, thereby justifying PB MRD monitoring at least at definite timepoints [2,44,54,75], perhaps with 0.01% cut-off value to define MRD negativity, even though using a >4-log reduction could also be considered reasonable [75]. Monitoring MRD in PB samples also offers other advantages, such as decreased procedural morbidity and the possibility of more frequent testing [8].

## 5. *NPM1* Mutation Levels at AML Diagnosis

In most series, *NPM1*-mutated transcript levels at diagnosis did not correlate with the type of *NPM1* mutation or with other clinical characteristics, namely age, sex, WBC count, BM blast count, lactate dehydrogenase level, ELN risk stratification or *FLT3* gene mutational status [7,9,12,19,26]. On the contrary, Balsat et al. found higher median *NPM1*-mutated baseline levels in cases with WBC count greater than 50 × 10^9^/L or with *FLT3*-ITD positivity, whereas no significant correlation was documented with age, karyotype or other concurrent gene mutations [29]. However, it should be noted that pretreatment *NPM1*-mutated expression levels detected by RQ-PCR did not influence CR rate after induction chemotherapy [19], CIR [19] and survival parameters, such as EFS and OS [7,12,19,26]. Conversely, Patel et al. recently reported targeted sequencing data from 109 patients with *NPM1*-mutated AML to retrospectively evaluate the potential significance of *NPM1* variant allele frequency (VAF) at diagnosis, comutations and clinical features on patient outcomes [76]. Interestingly, high *NPM1* VAF (≥0.44) observed on NGS correlated with shortened median OS (12.1 months versus not reached, *p* < 0.0001) as well as median EFS (7.5 versus 65.4 months, *p* < 0.0001). High *NPM1*-mutated allele burden at diagnosis was an independent predictor of unfavorable clinical outcomes, particularly in patients who received HSCT in first CR and in the subgroup of subjects with concomitant *DNMT3A* mutations. Because of BM multilineage involvement of *NPM1* mutations not being restricted to cells with blast morphology [12,16,20], the VAF could in fact reveal the true clonal disease burden. Higher *NPM1* mutant allele burden may be less amenable to eradication by intensive chemotherapy, potentially resulting in a higher likelihood of MRD persistence. Alternatively, high *NPM1*-mutated allele burden may indicate the presence of disease in different hematopoietic cell populations, belonging to the *NPM1*-mutated leukemic clone regardless of blast morphology, which could be intrinsically resistant to chemotherapy and could subsequently foster leukemia relapse, resulting in adverse clinical outcomes [76].

## 6. Sequential MRD Monitoring and Molecular Prediction of Relapse

Several studies have investigated whether *NPM1*-mutated MRD monitoring during follow-up of AML patients in CR after the end of intensive treatments could foresee hematologic relapse [31,33,34,35,37,38,39,40,44,47,52,54,61,65]. Notably, post-treatment serial testing may portend morphologic recurrence either when, in previously MRD-negative patients, *NPM1*-mutated transcripts again become detectable or, alternatively, when rising MRD levels are observed in patients with persistent low-level MRD positivity [75]. Ommen et al. documented that kinetics of molecular relapse was markedly different among AML with *NPM1* mutations or with rearrangements such as *PML-RARA, RUNX1-RUNX1T1* or *CBFb-MYH11*, and subsequently developed a mathematical model to predict the time elapsing between molecular and hematologic relapse [38]. According to this model, AML positive for *CBFb-MYH11* displayed a slower clone regrowth than AML with other molecular signatures, including *NPM1* mutations [38,77]. In many patients who previously obtained molecular CR, reoccurrence of *NPM1*-mutated transcripts may be detectable by molecular techniques over 2–3 months before overt morphologic relapse occurred [1,31,33,34,35,37,38,39,40,44,47,52,54,61,65], as summarized in Table 2, with significant differences observed between patients with concurrent *FLT3*-ITD positivity or negativity (65 versus 120 days, respectively) [38]. Furthermore, median time since molecular to hematologic relapse could also have a broad range, as documented by Kronke et al. (median 2.6 months, range 0.4 to 23.6) [40], and may significantly vary according to the *NPM1*-mutated/*ABL1* cut-off value considered for MRD positivity, as shown in the study Shayegi et al. [44]. Finally, Ivey et al. observed a longer time to overt relapse if molecular MRD was first detected in BM rather than in PB samples (133 versus 87 days, respectively) [54]. These data collectively suggest that serial molecular MRD assessment during follow-up may provide a timely prediction of impending morphologic relapse in *NPM1*-mutated AML patients, potentially allowing pre-emptive therapeutic approaches, preferably in the context of clinical trials [1,75,77].

## 7. MRD Levels and Allogeneic Hematopoietic Stem Cell Transplant in *NPM1*-Mutated AML

The use of alloHSCT in patients with *NPM1*-mutated AML is still controversial. In the retrospective study by Rollig et al., a significant improvement in RFS was documented in the donor group, indicating a beneficial effect of alloHSCT in *NPM1*-mutated AML patients in first CR, also observed in the favorable subgroup without *FLT3*-ITD [78]. Even in the absence of a significant difference in OS, most likely as a result of the fact that *NPM1*-mutated AML patients experiencing relapse often achieved response to salvage treatments, alloHSCT could represent a valuable consolidation option for *NPM1*-mutated AML patients in first CR if a sibling donor is available, especially in young patients, with a relatively low risk of non-relapse mortality [78]. Unfortunately, prospective MRD monitoring was not performed within this trial [78]. Notably, several subsequent studies investigated the prognostic significance of pre-transplant *NPM1*-mutated MRD levels (Table 2) [52,55,57,60,61,63,65,67]. In detail, Karas et al., among 60 *NPM1*-mutated AML patients undergoing alloHSCT, identified a significantly negative prognostic impact on EFS and OS, only for age above 63 years and for pre-transplant MRD positivity, immediately before starting the conditioning regimen, with cut-off 0.1% [52]. The estimated probabilities of 3-year relapse, EFS and OS were 6%, 72% and 75%, compared with 48%, 35% and 40% for patients with lower and higher levels of MRD, respectively [52]. Kayser et al. reported on 67 *NPM1*-mutated AML patients, receiving alloHSCT in either first (31 cases) or second (20 cases) CR or with refractory disease (16 cases) [55]. Overall, patients transplanted in CR had significantly longer OS as compared to patients with refractory disease. However, for patients undergoing alloHSCT in CR, there was a highly significant difference in OS between pre-transplant MRD-negative and MRD-positive cases, with estimated 5-year OS rates of 89% versus 40%, respectively. Interestingly, these latter patients had an OS rate comparable to that observed in patients receiving alloHSCT with refractory disease (38%). According to Kayser et al., pre-transplant *NPM1*-mutated MRD positivity (>1%) was a significant predictor of poor outcome after alloHSCT, independently of other variables, such as *FLT3*-ITD mutational status, BM blast count at diagnosis and age [55]. More recently, Bill et al. applied highly sensitive ddPCR to quantify pre-transplant *NPM1*-mutated MRD in 51 patients [61]. The 2-year CIR was 64.7% versus 6%, translating into OS rate 38.8% versus 71.7% in pre-transplant MRD-positive and MRD-negative cases, respectively. Thus, pre-transplant MRD persistence independently predicted adverse outcomes also in this cohort, including a large proportion of older patients (86.3% of cases, with median age 62 years), mainly receiving non-myeloablative conditioning regimens [61]. Interestingly, the recommendation to perform alloHSCT only based on other known prognostic factors, without considering pre-transplant *NPM1*-mutated MRD levels, is currently questionable [52,55,61]. According to Balsat et al., pre-transplant MRD assessment demonstrated that a survival benefit from alloHSCT was not seen for patients with >4-log *NPM1*-mutated MRD clearance, whereas it was maintained for those with insufficient MRD clearance, suggesting the relevant use of MRD evaluation in transplant selection, to be further validated in clinical studies [57,75]. However, it is so far uncertain whether patients with persistent MRD positivity could benefit from additional chemotherapy cycles before alloHSCT or, alternatively, from intensification of conditioning regimen, whenever possible. Prospective clinical trials are warranted to identify treatment options to improve the prognosis of *NPM1*-mutated AML patients resulting MRD-positive prior to alloHSCT [61].

Furthermore, since the first observation in a small patient cohort by Bacher et al. [34], post-transplant *NPM1*-mutated MRD monitoring has been shown to be predictive of relapse in several large retrospective series [35,36,44,63,65]. Schnittger et al. reported a significant effect on survival of *NPM1* mutation levels, with relevant threshold 0.1%, for samples analyzed between days 61 and 120 and between 121 and 365 after alloHSCT [35]. Similarly, Shayegi et al. documented that an increasing MRD burden exceeding the threshold of 10% *NPM1*-mutated/*ABL1* on RQ-PCR assay was strongly associated with an increased risk of relapse, therefore resulting in significantly worse outcomes in terms of DFS and OS [44]. Most recently, Zhou et al. retrospectively investigated the application of NGS to improve prediction of post-alloHSCT relapse in 59 adults with *NPM1*-mutated AML [63]. While pre-transplant MFC testing identified a subset of high-risk patients in whom additional therapy before HSCT could potentially be useful; high-sensitivity NGS analysis of *NPM1*-mutated around day 28 after alloHSCT predicted morphologic relapse in 15 of 18 cases (83%). Peri-transplant MRD evaluation, improving the risk assessment for relapse, may identify high-risk patients who could benefit from pre-emptive treatments after alloHSCT [63]. Moreover, Delsing Malmberg et al. reported that MRD analysis using targeted deep sequencing 3 months after alloHSCT was a strong independent predictor of both relapse risk and OS in adult *NPM1*-mutated AML patients [65]. Specifically, the 3-year OS was 20% ± 17.9% versus 88.6% ± 7.8% (*p* < 0.001) for MRD-positive and MRD-negative patients, respectively [65]. The above-presented data clearly suggest that *NPM1*-mutated AML patients undergoing alloHSCT should be carefully monitored by MRD assays after transplant in order to early detect imminent morphologic relapse [55,61]. However, the use of early pre-emptive strategies, namely tapering of immunosuppression, donor lymphocyte infusions, administration of hypomethylating agents or other targeted drugs, in patients with persisting or re-occurring *NPM1*-mutated MRD positivity after alloHSCT, are not currently standardized [55,61,75,79]. Based upon the pivotal RELAZA phase 2 study, which evaluated the role of 5-azacitidine in 20 either AML or myelodysplastic syndrome (MDS) patients with MRD positivity after alloHSCT [80], Platzbecker et al. recently reported the results of RELAZA2 trial, which investigated MRD monitoring in 205 patients with either advanced MDS (27 cases) or AML (178 cases, including 31 with *NPM1* mutations), in CR after either conventional chemotherapy only (58 cases) or consecutive alloHSCT (147 cases) [81]. In 53 patients who became MRD-positive at a median of 100 days after start of screening, pre-emptive 5-azacitidine was administered, resulting in overall response rates of 71% and 48% in patients with and without antecedent alloHSCT, respectively. At a median follow-up of 13 months, OS rate was 76%, suggesting that MRD-guided therapy with an hypomethylating agent may prevent or substantially delay morphologic relapse in high risk MDS or AML patients [81]. Moreover, Sockel et al. pre-emptively treated with 5-azacitidine 10 *NPM1*-mutated AML patients in first or second CR after intensive chemotherapy or autologous or allogeneic HSCT (3 cases), who experienced either molecular relapse or persistently detectable MRD positivity in sequential RQ-PCR analyses [82]. Molecular response, with at least 1-log decrease in the MRD level compared with pretreatment value, was obtained in 7 of the 10 patients. At a median follow-up of 10 months (range 2–12), a median of 5 cycles of 5-azacitidine were administered and the 7 patients remained in morphologic CR, suggesting a potential efficacy of 5-azaciditine in controlling *NPM1*-mutated MRD [82]. Although post-transplant early cellular or pharmacologic preemptive strategies to enhance graft-versus-leukemia effect or to eradicate persistent MRD have preliminarily demonstrated improved outcomes, their definitive clinical efficacy should be further investigated and determined in randomized clinical trials [79].

## 8. MRD Assessment in Elderly Patients with *NPM1*-Mutated AML

Incidence of AML is highest among older subjects, with a median age at disease onset of 67 years [83,84]. Despite the use of intensive chemotherapy in fit elderly patients, clinical outcomes remain generally dismal, with lower CR rates (around 40–50%) and few long-term survivors compared with younger patients [85,86]. Indeed, even when short-term benefits have been demonstrated, very few patients older than 60 years actually become long-term survivors, with OS rates reported to be <10% at 3 years and <5% at 5 years [85,86]. In addition to comorbidities, which commonly hamper intensive treatment approaches, it should be noted that the genetic profile of AML in the elderly is often unfavorable, with a high incidence of adverse-risk karyotypes and a lower frequency of good-risk molecular features [83]. Accordingly, *NPM1* mutations are found in only 20–25% of older AML patients, and in 35–40% of those with normal karyotype [83,87]. The possibility of obtaining CR remains relatively high (60–80%) in elderly *NPM1*-mutated AML patients [83,88]. However, while *NPM1*-mutated AML in younger adults is generally associated with favorable clinical outcomes, especially in the absence of *FLT3*-ITD, in older patients the risk of relapse is markedly higher (1-year CIR 71% in patients aged >65 years) and survival significantly lower (2-year OS 19%) [88]. In this disappointing clinical setting, *NPM1*-mutated MRD monitoring may potentially be useful for patients who obtain morphologic CR in order to define the best personalized post-remissional treatment approach and to early detect relapse [83]. However, although multiple series evaluating *NPM1*-mutated MRD also enrolled some patients aged >60 years (Table 1) [31,32,33,34,35,36,37,39,42,43,44,45,47,48,49,50,51,52,53,54,55,56,58,60,61,62,63,65,67,69,71], no study so far specifically focused on application and clinical significance of MRD monitoring in elderly patients with *NPM1*-mutated AML. Since the most predictive timepoints and MRD thresholds appear to be highly variable even between cohorts of younger adult patients, they cannot be easily applied to elderly subjects with poor prognosis and future studies are needed to further address these underexplored issues [83,89]. However, older AML patients frequently receive low to moderate-intensity treatments, mainly with hypomethylating agents, able to prolong survival even without the achievement of morphologic CR, thereby raising further controversies about the relevance of MRD studies in this clinical context [71,83].

## 9. Clonal Evolution and Loss of *NPM1* Mutation at AML Relapse

As expected for founder genetic lesions, *NPM1* gene mutation is generally stable throughout the course of the disease and has long been considered almost invariably present in patients experiencing AML relapse [14,90,91]. Notably, it has been detected at relapse even many years after the initial diagnosis, in patients experiencing more than one relapse and in relapses occurring in extramedullary sites [14,92,93]. However, after the first observation by Papadaki et al. that two among 21 (9.5%) *NPM1*-mutated AML patients experiencing relapse, lost *NPM1* mutation at leukemia recurrence [32], many groups investigating *NPM1*-mutated molecular MRD provided further information on stability of *NPM1* mutation at relapse, as summarized in Table 2. While in several cohorts, clonal AML evolution at disease recurrence was not observed [31,33,34,35,36,37,38,39,42,43,48,49,52,53,55,57,58,59,60,63,64,65,66,67,68,69], in the remaining studies the frequencies of patients experiencing AML relapse with undetectable *NPM1* mutations were extremely variable, ranging from 1% in the prospective trial by Ivey et al. [54] to 25% documented in smaller series [41,94]. Notably, cases with loss of *NPM1* mutation at disease reoccurrence were initially considered as secondary therapy-related myeloid neoplasms rather than true relapses from the previously found *NPM1*-mutated leukemic clone [14,32,40]. Interestingly, to address the role of clonal evolution in relapsed *NPM1*-mutated AML, Kronke et al. applied high-resolution SNP-array profiling and performed comprehensive gene mutation screening in 53 paired BM/PB samples obtained at diagnosis and relapse [95]. High stability between primary and relapse samples was observed for mutations in *DNMT3A* (97%), *IDH2* (92%) and *NPM1* (91%) genes, whereas *FLT3*-ITD (75%) and *IDH1* (75%) were less stable. Interestingly, *DNMT3A* mutations were consistently documented in primary and relapse samples from all the 5 cases with *NPM1* mutation loss at relapse, suggesting that *DNMT3A* mutation most likely preceded *NPM1* mutation in the pathogenesis of the disease and a common ancestral *DNMT3A*-mutated clone with *NPM1* wild-type gave rise to both primary and relapsed AML [95]. It should be noted that MRD monitoring based on RQ-PCR for *NPM1* mutations may be hampered in the rare cases where relapse develops from an ancestral *NPM1*-negative clone, probably arising from pre-existing non-malignant clonal hematopoiesis, therefore resulting in false-negative MRD results [81,94,95]. Furthermore, a switch of the *NPM1* mutation subtype from D to A has been recently described in a patient with late *NPM1*-mutated AML relapse, 8 years after the first diagnosis [96]. Clinical outcome of patients developing secondary AML with a switch of the *NPM1* mutation subtype may be misinterpreted by follow-up strategies only focusing on rising MRD levels alone, which assume recurrence of the *NPM1* mutation subtype previously documented at initial diagnosis [96]. These rare circumstances demonstrated potential limitations of RQ-PCR in MRD monitoring.

## 10. Newer Molecular Technologies to Assess MRD in *NPM1*-Mutated AML

A few studies have so far investigated the potential clinical application of newer molecular methods, namely ddPCR [27,48,59,61] and NGS [41,46,56,60,62,63,65,68,69,73], to monitor MRD in *NPM1*-mutated AML patients. As discussed above, at least 55 frameshift insertion *NPM1* mutations have so far been recognized, and several other different gene lesions are theoretically possible, rendering relative quantification of rare *NPM1* mutations by conventional RQ-PCR highly challenging [59]. Limitations of the application of RQ-PCR-based MRD assays are their dependence on specific gene mutations, requiring individual reference standard curves based on target serial dilutions, with commercial plasmid standards being widely available for only the three most common *NPM1* mutation types [59,77]. ddPCR, a high-throughput technology, instead generates absolute quantification, can clonally amplify target nucleic acids, and does not require a reference standard curve [10,77]. More specifically, in ddPCR assays the sample is separated in a large number of partitions, and PCR reactions are compartmentalized to single oil droplets, which are then analyzed for presence or absence of a target sequence based on fluorescence. Through the use of Poisson statistics, the starting concentration of the target sequence is measured based on the number of individual reactions in which it is found to be present [2,10,97]. According to the first few clinical experiences in small *NPM1*-mutated AML patient series, ddPCR demonstrated excellent sensitivity and agreement with RQ-PCR, also allowing for the detection of a variety of rare *NPM1* mutation subtypes (Table 1 and Table 2) [48,59]. Notably, Mencia-Trinchant et al. created oligonucleotides with degenerate sequence at known insertion sites and combined these massively multiplex pools of insertion-specific primers into a single assay, which may selectively detect virtually all known and potential *NPM1* mutations, but not wild-type *NPM1* sequences [59]. Even if the precision of ddPCR assay, combined with its broad applicability also in patients ineligible for RQ-PCR MRD assessment, should be recognized, it should also be noted that RQ-PCR actually still performs more than adequately for MRD detection in most *NPM1*-mutated AML patients, is less expensive, and many clinical laboratories already possess the equipment and technical expertise for this molecular analysis [98]. In addition, primer mixes to detect multiple *NPM1* mutation could also be used in RQ-PCR assays [35,37,54,99], while Mencia-Trinchant et al. do not report whether their primer mix can potentially be utilized in RQ-PCR assays [59]. However, the multiplexing of ddPCR could allow a future wider use of this molecular tool in *NPM1*-mutated MRD monitoring, after further validation in prospective clinical trials, at least in cases showing rarer mutations [97].

Furthermore, multi-gene NGS-based MRD monitoring has recently been applied to AML patients, including those showing *NPM1* mutations (Table 1 and Table 2) [41,46,56,60,62,63,65,68,69,73]. NGS technologies, which allow parallel and repeated sequencing of millions of small DNA fragments across many loci in order to evaluate either a few genes or an entire genome, may overcome some of the limitations of single genes analysis based on RQ-PCR assays [10,75,77,100]. The ability of NGS platforms to study large numbers of mutated genes could help to trace the evolution of malignant clones [77]. However, the genetic clonal heterogeneity at AML diagnosis and during the course of the disease could, conversely, complicate the MRD monitoring, because the predominant leukemic clone at presentation may not be the same clone that determines clinical relapse and mortality [77]. Moreover, intratumoral heterogeneity relies not only on the presence of different somatic mutations in distinct leukemic subclones, but also on heterogeneity of transcriptional and epigenetic states [101,102,103]. Unlike population-level approaches, single-cell RNA-sequencing platforms enable transcriptomic analysis of an individual cell. Through the combination of high-throughput sequencing and bioinformatic tools, single-cell RNA sequencing can detect more than 10,000 transcripts in one cell to distinguish cell subsets and dynamic cellular changes, therefore revealing intratumoral heterogeneity, with possible applications in identifying treatment-resistant leukemic cells and MRD monitoring [101,102,103]. Another significant limitation of NGS technology initially was the intrinsic error rate that limited its sensitivity for most single-nucleotide variants to 1–2% of all reads [1,104]. This notwithstanding, recent refinements of NGS technology, such as the introduction of random barcodes that anneal to target sequence fragments prior to amplification or unique molecular indexes, have eliminated the error rate of sequencing itself and markedly improved the assay sensitivity, which is actually comparable or even superior to that of RQ-PCR or MFC [75,105]. Thol et al. first conducted parallel assessment of MRD by NGS and RQ-PCR in a small series of *NPM1*-mutated AML patients, observing concordance in 95% of the 38 analyzed samples [41]. As mentioned above, Zhou et al. recently compared MRD testing by MFC and NGS before and after alloHSCT and showed that NGS was approximately 10-fold more sensitive than MFC, also demonstrating the clinical utility of high-sensitivity deep sequencing in risk assessment of relapse, especially after HSCT [63]. Moreover, Onecha et al. optimized and validated a high-sensitivity NGS method to detect and quantify *NPM1, IDH1/2* and *FLT3* mutated sequences, with survival analyses showing that MRD-positive status tested by NGS was associated with higher risk of relapse and death, whereas MRD-negativity at post-consolidation correlated with a longer DFS and OS [69]. Although NGS assay currently shows a similar sensitivity to that of RQ-PCR, it does not require oligonucleotides that hybridize specifically to a particular sequence, so all the nucleotides in the amplified region can be studied [69]. Consequently, the NGS test is capable of simultaneously detecting all *NPM1* mutation types in a single assay [41,46,56,65]. Therefore, the application of either NGS or multiplex ddPCR may be indicated for follow-up quantification of rare *NPM1* mutations subtypes, or alternatively in anecdotal cases of a switch of the *NPM1* mutation subtype during the course of the disease [69,96]. Despite these potential advantages, NGS technology is still computationally demanding, time-consuming and expensive, so that the ELN MRD Working Party suggests that NGS techniques for MRD measurement are actually best reserved for clinical trials for further validation [74,77].

## 11. Persistence of Pre-Leukemic Clones at AML Remission: What about *NPM1* Mutations?

In the seminal analysis of comprehensive genomic data on samples collected from 50 patients at AML presentation and remission after induction chemotherapy, Klco et al. documented persistent leukemia-associated mutations in at least 5% of BM cells at remission in 48% of cases, which were associated with increased relapse risk and reduced OS. Interestingly, contrary to other genes, *NPM1* mutations were cleared below the VAF 2.5% threshold at day +30 after chemotherapy in all the 18 *NPM1*-mutated AML analyzed cases [106]. Accordingly, Hirsch et al. reported, in a 69-AML-patient cohort, that the detection of two or more events in >0.4% of cells by NGS assay after one treatment course was strongly associated with lower survival in all cytogenetic profiles, especially in cases with intermediate-risk cytogenetics [107]. Furthermore, Rothenberg-Thurley et al. characterized paired pre-treatment and remission samples from 126 AML patients for mutations in 68 leukemia-associated genes. Fifty cases (40%) retained, based on NGS analysis, at least 1 mutation during remission at a VAF of ≥2%, with mutation persistence more frequently observed for *DNMT3A* (65%), *SRSF2* (64%), *TET2* (55%) and *ASXL1* (46%), whereas *NPM1* mutation was rarely persisting (1/57 patients, 2%) [108]. Interestingly, among 46 patients with *NPM1* mutation, there was a non-significant trend towards higher molecular MRD levels by RQ-PCR in patients with compared to those without persisting pre-leukemic lesions. A correlation was observed between persistence of pre-leukemic clones during first remission and inferior OS and higher incidence of relapse, which was abrogated, in this series, by alloHSCT, suggesting that mutation persistence may guide post-remission treatments [108]. More recently, further information on the prognostic role of different persisting molecular lesions in AML patients at remission has been provided [62,109]. Specifically, among 131 previously untreated AML patients, Morita et al. observed frequent persistence of somatic mutation at CR in genes that are often pre-leukemic and associated with clonal hematopoiesis of undetermined significance (CHIP), namely *DNMT3A*, *TET2, SRSF2, ASXL1* and *TP53*, whereas mutations in *NPM1* gene, transcription factors or receptor tyrosine kinase genes were often cleared [109]. Although the optimal VAF cut-off remains to be determined, patients who achieved mutation clearance with a VAF <1% at day +30 after therapy, had significantly better survival and lower risk of relapse in this series. These prognostic associations were more pronounced when CHIP-related gene lesions were removed from the analysis [109]. In a larger cohort of 482 AML patients, targeted NGS found persisting gene mutations in 51.4% of patients during CR, with VAF ranging from 0.02% to 47%. The detection of persistent mutations in *DNMT3A*, *TET2* and *ASXL1* genes (*DTA* mutations) was not correlated with an increased relapse rate. Only after exclusion of *DTA* mutations from the analysis, the documentation of molecular MRD display significantly higher relapse rates and lower survival rates, thereby confirming that persistent mutations in genes associated with age-related CHIP did not have prognostic value in adult patients within a 4-year time frame [62]. Overall, these studies provided information on the use of a genome-wide approach to evaluate the impact of persistent multiple gene mutations on treatment outcomes [62,106,107,108,109]. The monitoring of specific single-gene MRD levels, including quantification of *NPM1* transcripts generally absent in pre-leukemic clones, could potentially be integrated with evaluation of persisting pre-leukemic molecular lesions at remission. The combination of these two variables may ultimately provide complimentary prognostic information and it could potentially be argued that monitoring both leukemic and pre-leukemic clones after therapy could, in perspective, complement or even supersede pre-treatment genetic factors for AML prognostic stratification [108].

## 12. Conclusions

Even though several published clinical studies (Table 1 and Table 2) support the independent prognostic significance of molecular MRD monitoring in *NPM1*-mutated AML patients, and the Consensus Document from ELN MRD Working Party clearly indicates that patients showing *NPM1* mutations should have molecular assessment of MRD, we have discussed that several controversies have been raised, and further prospective clinical trials are warranted to standardize the optimal material, timepoints, molecular tools and cut-offs for a meaningful application of MRD monitoring to inform treatment decisions [31,74,110]. Caution is still needed when using MRD results alone, outside of a clinical trial, to make decisions with regard to treatment approaches with potentially high morbidity and mortality, mainly alloHSCT, in otherwise genetically favorable-risk AML patients [8]. Interestingly, the application of pre-emptive, MRD-directed treatment strategies, potentially including pharmacological agents targeting NPM1-mutated protein with induction of protein degradation or drugs inhibiting NPM1 cytoplasmic translocation, such as selinexor, which acts through inhibition of nuclear export and resulting in NPM1 protein nuclear relocalization, should be warranted and needs to be prospectively investigated [111,112,113].

## Figures and Tables

**Table 1:** MRD monitoring in *NPM1*-mutated AML series: review of the literature.

Reference/Type of Study	Number of Patients/Median Age (Years, Range)	Number of Samples (PB/BM)	Number of Samples per Patient (Median, Range)	Molecular Method/Material	*NPM1* Mutation Type	Sensitivity of the Assay
Gorello et al., 2006 [30]/retrospective	20/NA	NA (PB and/or BM samples at diagnosis and/or at different timepoints)	NA (13 patients analyzed at diagnosis and post-induction). MRD kinetics during follow-up of 3 representative patients is reported	RQ-PCR/cDNA (5 cases), DNA (15 cases)	A, B (cDNA); A, B, D, E, G, H (DNA)	10^−3^–10^−6^
Chou et al., 2007 [31]/retrospective	38/47 (17–87)	194 BM	5	RQ-PCR/DNA	7 different mutations	10^−5^
Papadaki et al., 2008 [32]/retrospective	51/58 (22–78)	154 (18 PB/136 BM)	NA (26 patients analyzed at diagnosis and at least at 2 timepoints during therapy; 27 patients analyzed at diagnosis and after induction therapy)	RQ-PCR/cDNA	A	10^−5^
Barragan et al., 2008 [33]/-	24/17 cases (71%) <60 yeas	97 (5 PB/92 BM)	NA	RQ-PCR/cDNA	A	10^−5^
Bacher et al., 2009 [34]/retrospective	13/47 (20–66)	139 (PB/BM)	7 (2–25)	RQ-PCR/DNA	A, B	10^−4^–10^−6^
Schnittger et al., 2009 [35]/retrospective	252/59 (20–79)	1227 (28 PB at diagnosis/1199 BM)	4 (2–16)	RQ-PCR/cDNA	17 different mutations	10^−4^–10^−6^
Stahl et al., 2010 [36]/retrospective	25/53 (21–73)	76 (38 PB/38 BM)	1–2	RQ-PCR/DNA	A	10^−4^–10^−6^
Dvorakova et al., 2010 [37]/retrospective	25/51 (43–75)	1026 (339 PB/687 BM)	28 (11–68)	RQ-PCR/DNA	9 different mutations	10^−4^–10^−6^
Ommen et al., 2010 [38]/retrospective	180 (54 in HR)/NA	193 CCR and 70 relapse samples	NA	RQ-PCR/cDNA	NA	10^−4^–10^−6^
Kristensen et al., 2011 [39]/retrospective	20/61 (41–76)	204	NA	RQ-PCR/DNA	A	2.4 × 10^−5^
Kronke et al., 2011 [40]/retrospective	245/49 (19–61)	1682 (410 PB/1272 BM)	NA	RQ-PCR/cDNA	6 different mutations	10^−5^–10^−6^
Thol et al., 2012 [41]/retrospective	10/NA (adult patients)	45	NA	NGS, RQ-PCR/DNA, cDNA	3 different mutations (8 cases A, 1 case D, 1 case atypical)	10^−4^
Abdelhamid et al., 2012 [42]/retrospective	20/55 (27–69)	116 (20 at diagnosis, 96 follow-up samples, namely 55 PB and 41 BM)	4.5 (2–11)	RQ-PCR/DNA	3 different mutations (A, B, insCACG)	10^−4^–10^−5^
Schiller et al., 2012 [43]/retrospective	30 (among 54 *FLT3*-ITD+ patients)/62	NA	NA	RQ-PCR/cDNA	NA	10^−4^–10^−6^
Shayegi et al., 2013 [44]/retrospective	155/51 (20–79)	1750 (817 PB/933 BM)	NA	RQ-PCR/DNA	A, B, D	10^−5^
Jeziskova et al., 2013 [45]/retrospective	6 (among 8 patients with *IDH2* mutations)/57 (53–72)	60 (17 PB/43 BM)	3–14	RQ-PCR/DNA	A, B	10^−4^–10^−6^
Salipante et al., 2014 [46]/retrospective	6/NA	22 BM	2–6	NGS/DNA	No need for mutation-specific probes	10^−5^
Hubmann et al., 2014 [47]/retrospective	158/57 (18–80)	588 BM	NA	RQ-PCR/cDNA	A, B, D	10^−6^
Bacher et al., 2014 [48]/retrospective	99/NA	498	4 (1–28)	digital PCR/cDNA	37 different mutations	10^−4^–10^−5^
Lambert et al., 2014 [49]/prospective	77 patients with *NPM1* mutation/61 (57–65)	250 (125 PB/125 BM)	NA	RQ-PCR/cDNA	A, B, D	10^−5^
Debarri et al., 2015 [50]/retrospective	31/60 (23–70)	94	NA	RQ-PCR for *NPM1*; NGS for *IDH1/2* and *DNMT3A*/cDNA	A, B, D	10^−5^
Pettersson et al., 2016 [51]/-	19/64 (28–78)	63 (2 PB/61 BM)	1–9	RQ-PCR/DNA	A	10^−5^
Karas et al., 2016 [52]/retrospective	60/54 (30–66)	60 BM	1 (pre-HSCT)	RQ-PCR/cDNA	A, B, D	NA
Alizad Ghandforoush et al., 2016 [53]/retrospective	11/42 (28–63)	71 (PB/BM)	NA	RQ-PCR/DNA	A	10^−5^
Ivey et al., 2016 [54]/prospective	346 in preliminary development phase, 91 in validation cohort/50 (6–68)	2569 in first phase (1667 PB/902 BM)	6	RQ-PCR/cDNA	27 different mutations	10^−5^
Kayser et al., 2016 [55]/retrospective	67/55 (21–70)	406 (BM) at different timepoints	pre-transplant MRD was available for 39/51 (76.4%) patients in CR at HSCT (22 cases, 56% MRD-positive)	RQ-PCR/cDNA	A, B, D	10^−5^–10^−6^
Malmberg et al., 2017 [56]/retrospective	17 (3 *NPM1*-mutated patients)/39 (2–71)	NA	NA	RQ-PCR, NGS/DNA	A	10^−5^
Balsat et al., 2017 [57]/retrospective	152/49 (21–61)	304 (PB/BM) at diagnosis and 270 post-induction (135 PB/135 BM)	samples obtained at diagnosis and after induction CHT	RQ-PCR/cDNA	A, B, D	10^−5^
Schieppati et al., 2017 [58]/-	68/56 (27–74)	NA	4 PB/BM samples (at diagnosis, TP1 at CR, TP2 post-consolidation, TP3 post 1st cycle of Ara-C)	RQ-PCR/cDNA	NA	10^−4^–10^−5^
Mencia-Trinchant et al., 2017 [59]/-	3/-	NA	Sequential determination of MRD levels	ddPCR/cDNA	multiplex assay effective in a range of diverse common and rare *NPM1* mutations (14)	10^−4^–10^−5^
Getta et al., 2017 [60]/retrospective	104 (10 with *NPM1* mutation)/58 (21–78)	58 BM at diagnosis, 83 BM before HSCT for NGS	NA	NGS, MFC/DNA	2 different mutations	10^−4^
Bill et al., 2018 [61]/retrospective	51/62 (33–74)	51 (40 PB/11 BM)	samples collected directly before HSCT	ddPCR/cDNA	A, D	10^−4^
Jongen-Lavrencic et al., 2018 [62]/retrospective	430 (168 with *NPM1* mutation)/51 (18–66)	482 PB/BM samples at diagnosis, 430 BM samples after treatment	2 (at diagnosis and in CR)	NGS, MFC/DNA	NA	10^−4^
Zhou et al., 2018 [63]/retrospective	59/57(21–79)	104 BM	pre-HSCT and post-HSCT	NGS, MFC/DNA	-	10^−4^
Zappasodi et al., 2018 [64]/retrospective, real-life study	201 (116 with *NPM1* mutation)/58	NA	Availability of samples during treatment and follow-up was variable.	RQ-PCR/cDNA	NA	10^−4^–10^−5^
Delsing Malmberg et al., 2018 [65]/retrospective	29/49 (18–66)	83 (6 PB/77 BM)	3 (at diagnosis, before and after HSCT)	NGS/DNA	all recurrent insertion mutations in *NPM1* exon 12 (mutation A in 25 cases)	10^−4^
Kapp-Schwoerer et al., 2018 [66]/retrospective	611/18–60	6339 (2812 PB/3527 BM)	NA (samples analyzed at diagnosis, during treatment and follow-up)	RQ-PCR/cDNA	NA	10^−5^–10^−6^
Caprioli et al., 2018 [67]/retrospective	27/57 (23–65)	27 BM	1 (pre alloHSCT)	RQ-PCR/cDNA	NA	10^−4^
Patkar et al., 2018 [68]/retrospective	83/NA	NA	NA	NGS/DNA	12 different mutations	10^−5^
Onecha et al., 2018 [69]/retrospective	63 (57 with *NPM1* mutation)/54 (42–66)	106 BM (51 after induction, 55 post consolidation CHT)	2	NGS/DNA	A	10^−5^
Petrova et al., 2018 [70]/retrospective	90 (22 positive for *IDH1/2* mutations, 11 with *NPM1* mutation)/61 (22–82)	149 BM	NA (90 at diagnosis, 22 after induction, 37 during follow-up)	RQ-PCR for *NPM1* mutations/cDNA	A	NA
Prata et al., 2018 [71]/retrospective	34 with newly diagnosed *NPM1*-mutated AML/77 (55–85)	MRD assessment available on BM samples in 6 patients	NA	RQ-PCR/NA	A, B, D	NA
Ottone et al., 2018 [72]/retrospective	556 de novo AML (177 with *NPM1* mutation)/49 (16–89)	NA (BM samples at diagnosis, during follow-up, at relapse)	NA (*NPM1*-mutated transcripts monitoring in 51 cases)	RQ-PCR/cDNA	A	10^−5^
Gaksch et al., 2018 [73]/retrospective	34 cytogenetically normal AML (16 cases with *NPM1* mutation)/47 (22–79)	34 BM or PB at diagnosis, 27 BM samples in remission	2 (at diagnosis and after at least one consolidation therapy)	NGS/DNA extracted from BM slides	Multiplex analysis of 19 genes, including *NPM1* mutational hotspots	10^−2^

MRD, minimal/measurable residual disease; AML, acute myeloid leukemia; PB, peripheral blood; BM, bone marrow; NA, not available; RQ-PCR, real-time quantitative polymerase chain reaction; cDNA, complimentary DNA; HR, hematological relapse; CCR, continuous complete remission; NGS, next-generation sequencing; HSCT, hematopoietic stem cell transplantation; CR, complete remission; TP, timepoint; ddPCR, digital droplet PCR; MFC, multiparametric flow cytometry; CHT, chemotherapy.

**Table 2:** Clinical impact of MRD monitoring in *NPM1*-mutated AML patients.

Reference	Intensive CHT/HSCT (No. of Patients)	Significant MRD Threshold	Prognostic Timepoints	Correlation with Other Molecular Markers	Clonal Evolution	Median Time since Molecular to Morphologic Relapse (Range)	Clinical Relevance
Gorello et al., 2006 [30]	Yes/NA	NA (3/5 cases with MRD <1% long-term survivors)	NA	NA	NA	NA	- *NPM1*-mutated copies closely correlated with clinical status and predicted impending hematologic relapse in 2 cases
Chou et al., 2007 [31]	38 Yes/11 alloHSCT	0.1%	End of consolidation; follow-up	*FLT3*-ITD worsened RFS	No	4.9 months (1–12.3)	- Any rise of mutant signals during follow-up increased relapse risk - MRD < 0.1% predicted longer RFS and OS - Failure to achieve 2 logs reduction after consolidation predicted shorter RFS and OS
Papadaki et al., 2008 [32]	50 Yes/11 alloHSCT	NA (median log_10_ reduction of 2.48 post induction correlated with response to therapy)	NA	NA	In 2/21 relapses (9.5%) *NPM1* mutation was lost.	NA	- In selected patients, it was possible to correlate the changes of the *NPM1* mutation A levels with the clinical course of the disease
Barragan et al., 2008 [33]	24 Yes/NA	NA (in 19 patients in CCR a median 3% MRD after induction was shown. Median MRD level after consolidation 0.3%	NA	Expression levels of *WT1* and *NPM1* showed strong positive correlation.	No	MRD increase 1 to 5 months before relapse in 4/6 cases	- MRD negativity or maintenance of very low levels of *NPM1* mutation in patients in continuous CR - Increase in *NPM1* transcripts before or at the time of relapse
Bacher et al., 2009 [34]	-/13 alloHSCT	NA	All 4 patients (29%) with persistent MRD positive after HSCT relapsed	Correlation with molecular chimerism.	No	24 days (12–38)	- After HSCT 10/14 cases (71%) PCR-negative, of which 4 achieved stable CR. - MRD increase preceded morphologic relapse
Schnittger et al., 2009 [35]	252 Yes/53 alloHSCT	0.01% during 1st line treatment.0.1% after HSCT and during 2nd line treatment.	- early assessment (days 18–60) - days 60–121 - days 121–365 - longer than 1 year after start of treatment	*FLT3*-ITD prognostic factor affecting EFS	No	62 days (15–221)	- MRD for *NPM1* mutation assessed at 4 different time intervals after start of therapy (mainly after day 60) is an independent and highly predictive parameter for EFS.
Stahl et al., 2010 [36]	-/25 alloHSCT	NA	Post-HSCT follow-up	High rate of congruent results with chimerism analysis	No	NA	- Concordant results in BM and PB in 60% of sample pairs. - Cases with <0.01% MRD in BM were negative in PB. - Higher MRD levels in BM (>0.2%) predicted PB positivity
Dvorakova et al., 2010 [37]	25 Yes/4 alloHSCT	Reappearance of *NPM1* mutation or one order NCN increase in patients with persistent positivity at any timepoint.	NA	NA	No	97 days (12–141)	- Molecular relapse preceded hematological relapse in 80% of evaluable patients. - Strong correlation between PB and BM samples
Ommen et al., 2010 [38]	NA/NA	0.005% (threshold to define molecular relapse)	NA	More rapid MRD growth in *FLT3*-ITD+ cases	No	120 days without *FLT3*-ITD, 65 days in *FLT3*-ITD+ cases	- Mathematical model to determine the frequency of relapse detection - Sampling every 4 and 6 months suggested in *FLT3*-ITD+ and negative cases, respectively
Kristensen et al., 2011 [39]	20 Yes/NA	NA	Reoccurrence of *NPM1* mutation at any time was associated with relapse.	*NPM1* mutation is superior to *WT1* expression levels as MRD marker.	No (*NPM1* mutation stability. Karyotype evolution in 56% of relapses).	46 days (20–182)	- All relapses were associated with high levels of *NPM1* mutation - Detectable *NPM1* mutation following a CR period was accompanied by morphologic relapse in all cases
Kronke et al., 2011 [40]	245 Yes/80 alloHSCT	2%	- -MRD negativity after 2 induction cycles - -After completion of therapy - -During follow-up	*FLT3*-ITD significant factor for inferior survival.	In 5 patients *NPM1* mutation was not detectable at relapse (9% of evaluable relapse samples)	2.6 months (0.4–23.6)	- Observation of higher *NPM1*-mutated transcript levels after double induction or after completion of consolidation therapy was a significant factor for higher risk of relapse and death. - Serial post-treatment MRD assessment allowed early detection of relapse
Thol et al., 2012 [41]	10 Yes/NA	NA (mean allelic ratio at diagnosis 0.37, range 0.29–0.46)	NA	NA	*NPM1* mutation not detectable in 1/4 patients at relapse.	NA	- Parallel assessment of MRD by NGS and RQ-PCR was concordant in 95% of analyzed samples - NGS as a potentially highly flexible and reliable tool to assess MRD
Abdelhamid et al., 2012 [42]	20 Yes/NA	NA	After induction therapy	Similar kinetics of *FLT3*-ITD, *NPM1* and *WT1* expression for predicted clinical trend.	No	NA for *NPM1* mutation	- The 3 MRD markers tested showed comparable kinetics in 17/20 (85%) cases
Schiller et al., 2012 [43]	54 (30 *NPM1*-mutated) Yes/7 alloHSCT	NA	NA (samples collected at diagnosis, during treatment and follow-up)	MRD for *FLT3*-ITD as sensitive as other MRD parameters as *NPM1* mutations or *MLL*-PTD	NA	NA	- MRD negativity predicted lasting remission independent of alloHSCT or non-alloHSCT - *FLT3*-ITD analyses equivalently sensitive in PB samples
Shayegi et al., 2013 [44]	155 Yes/40 alloHSCT	-MRD level >1% after conventional CHT-MRD level >10% after alloHSCT	After intensive CHT and after HSCT	Prognostic role of MRD remained significant after adjustment for *FLT3*-ITD status	No	- 121 days (70–172) for MRD > 1% - 66 days (34–98) for MRD > 10%	- Rising of MRD was associated with increased risk of relapse. - DFS and OS analyses revealed significantly worse outcomes in patients with rising MRD levels
Jeziskova et al., 2013 [45]	8 Yes/4 alloHSCT	NA	NA	Concordance of quantitative detection of *IDH2* and *NPM1* mutations, except for on case.	NA	NA	- In 5/6 patients, the kinetics of *IDH2* quantification was nearly identical to the kinetics of *NPM1* mutations
Salipante et al., 2014 [46]	6 Yes/NA	NA	NA	NA	In 2 patients genetically distinct *NPM1* tumor subclones were detected	NA	- As a proof of principle, NGS documented MRD in all samples deemed negative by flow cytometry, without the need for mutation-specific probes
Hubmann et al., 2014 [47]	158 Yes/30 alloHSCT	cut-off ratio 0.01 and 3-log reduction	After induction CHT	Prognostic role of MRD regardless of ELN risk stratification.	*NPM1* mutation not detectable in 3 of 45 (6.7%) patients at relapse	58 days (20–98)	- Assessment of MRD levels after induction was significant to identify patients in CR with high risk of relapse. There was also a trend for OS
Bacher et al., 2014 [48]	99 Yes/NA	0.01%	- days 18–60 - days 61–120 - days 121–365 - >day 365	NA	No	NA	- *NPM1* mutation levels by digital and conventional RQ-PCR significantly correlated a diagnosis and during follow-up - digital PCR threshold of 0.01% prognostically relevant.
Lambert et al., 2014 [49]	77 Yes/NA	0.1% (in BM samples)	- after induction CHT - at the end of treatment	After adjustment for *FLT3*-ITD status, the effect of *NPM1* MRD appeared to be similar.	No	NA	- *WT1* MRD associated with increased hazard of relapse and shorter OS from CR. - Positive *NPM1*-mutated MRD predicted higher risk of relapse, but did not influence OS - Achievement of negative *NPM1* mutation MRD significantly more frequent in GO arm
Debarri et al., 2015 [50]	31 Yes/NA	0.1%	- post induction CHT - post first and second consolidation courses	Analysis of correlation between *NPM1* mutations and *IDH1/2* or *DNMT3A* mutations	One patient with *IDH2* mutation developed a *NPM1*-negative MDS	NA	- *IDH1/2* mutational status by NGS predicted relapse or disease evolution in 100% cases - *DNMT3A* mutations not correlated with disease status (a preleukemic clone persisted in 40% of cases in CR)
Pettersson et al., 2016 [51]	15 Yes/5 HSCT	0.1%	During follow-up	NA	One of 8 relapsing patients developed a *NPM1*-negative relapse	NA	- RQ-PCR of NPM1 A type mutation was more sensitive and reliable than MFC for determination of MRD
Karas et al., 2016 [52]	-/60 alloHSCT	0.1%	pre-transplant (less than 1 week prior to start conditioning regimen)	*FLT3*-ITD positivity had no adverse effect on HSCT outcome in patients with AML with *NPM1* mutation in CR	No	4 months (3–13) from HSCT to relapse, especially in patients with high preHSCT MRD	- older age and pre-transplant MRD had independent prognostic impact on EFS and OS - 3-year relapse rate, EFS and OS were 6%, 72%, 75% with low-level MRD and 48%, 35%, 40% in patients with higher MRD levels
Alizad Ghandforoush et al., 2016 [53]	11 Yes/6 alloHSCT	<5 log reduction	during follow-up	NA	No	NA	- Relapse occurred in 6 (54.5%) patients, whose *NPM1* mutation levels showed less than 5 log reduction after treatment
Ivey et al., 2016 [54]	346 + 91 Yes/82 alloHSCT	0.01%	after second CHT cycle (PB samples)	Presence of *FLT3*-ITD or *DNMT3A* mutations did not provide additional prognostic information	*NPM1* mutations detectable in 69/70 (99%) patients at relapse	133 days (BM), 87 days (PB)	- Persistence of *NPM1* mutated transcripts, observed in 15% of patients after second CHT cycle, associated with greater risk of relapse after a 3-year follow-up and lower survival rate (24% vs. 75%) - MRD as the only independent prognostic factor for death
Kayser et al., 2016 [55]	-/67 alloHSCT	1%	prior to alloHSCT	*FLT3*-ITD status had no impact on prognosis	No	NA	- Significant difference in OS after alloHSCT between pre-transplant MRD-positive and MRD-negative patients (estimated 5-year OS rates 40% vs. 89%) - Outcome of patients with preHSCT MRD positivity as poor as that of cases transplanted with refractory disease
Malmberg et al., 2017 [56]	NA/NA	0.001%	NA	*CPS1, FAM193A, ITGB7*	NA	NA	- For mutation load of *NPM1*, good correlation between results from deep sequencing and RQ-PCR - Targeted deep sequencing more sensitive for MRD quantification than MFC
Balsat et al., 2017 [57]	152 Yes/44 alloHSCT	4-log reduction in PB MRD	post-induction CHT cycle	Abnormal karyotype, *FLT3*-ITD, PB MRD associated with higher relapse incidence and shorter OS.	No	NA	- Patients without early MRD reduction had higher incidence of relapse and shorter OS - In patients with non favorable AML, HSCT improved outcomes only in case of <4-log reduction PB-MRD
Schieppati et al., 2017 [58]	68 Yes/9 alloHSCT	0.5%	TP1 (BM), TP3 (PB)	*FLT3*-ITD did not impact on relapse risk	No	NA	- Molecular MRD monitoring of crucial importance in detecting relapse at an early stage
Mencia-Trinchant et al., 2017 [59]	3 Yes/-	NA	NA	NA	No	NA	- Novel ddPCR technique composed of massively multiplex pools of insertion-specific primers that selectively detected virtually all *NPM1* mutations in a manner that was robust to clonal heterogeneity and did not require *NPM1* sequence information
Getta et al., 2017 [60]	-/104 alloHSCT	<5% VAF	before HSCT	NA	No	NA	- Mutations in *DNMT3A, TET2, JAK2* less likely to be cleared than *NPM1* (negative in 8/9 cases), *IDH1/2*, *FLT3-ITD* - MRD detected concurrently with MFC and NGS conferred the highest relapse risk
Bill et al., 2018 [61]	51 Yes/51 HSCT	0.01%	prior to HSCT	Adverse prognostic role of *NPM1* mutation MRD independent of other known prognostic markers	NA	101 days after HSCT (median time to relapse for all patient cohort)	- 17/51 (33.3%) patients were MRD positive before HSCT - The 2-year cumulative incidence of relapse was 64.7% vs. 6% translating into OS 38.8% vs. 71.7% in pre HSCT MRD+ and MRD-negative, respectively
Jongen-Lavrencic et al., 2018 [62]	430 (168) Yes/	2.5% allele frequency	samples obtained during a defined period of remission, between 21 days and 4 months after start of second treatment cycle	-	NA	NA	- mutations persisted in 51.4% of patients during CR - Persistent DTA mutations not correlated with increased relapse rate - Persistent non-DTA mutations conferred higher relapse rates and lower survival rates
Zhou et al., 2018 [63]	-/59 alloHSCT	0.01%	pre-HSCT and post HSCT (around day +28)	Peri-HSCT MRD independent prognostic factor	No	NA	- Before HSCT MRD detected by MFC was the most significant risk factor for relapse - NGS testing of *NPM1*-mutated post-HSCT improved the risk assessment of relapse
Zappasodi et al., 2018 [64]	201 Yes/4 alloHSCT	NA	MRD negativity at any time, during or after the end of 1st line treatment	NA	No	NA	- molecular CR obtained in 73.7% of *NPM1*-mutated cases - molecular CR at the end of treatment prognostic factor for DFS and OS in *NPM1*-mutated AML
Delsing Malmberg et al., 2018 [65]	-/29 alloHSCT	0.02%	pre and post-HSCT	MRD was an independent risk factor associated with clinical outcome	No	4.5 months (3.5–11) for MRD+; 7.7 months (7–32) for MRD-cases	- Post-HSCT deep sequencing MRD status was significantly associated with clinical outcome (3-year OS 20% vs. 89%)
Kapp-Schwoerer et al., 2018 [66]	611 Yes/162 alloHSCT	2%	post two CHT cycles (BM)	*NPM1* MRD negativity associated with lower relapse rate and better OS independent of *DNMT3A* and *FLT3*-ITD mutations	No	NA	- Outcome of patients who became MRD negative or remained positive but had 2017 ELN favorable risk profile was superior after high-dose AraC consolidation than after alloHSCT - MRD monitoring useful to inform post-remission therapy
Caprioli et al., 2018 [67]	-/27 alloHSCT	0.01%	pre-alloHSCT	NA	No	NA	- molecular analysis had higher sensitivity than MFC - Low-level or negative MRD associated with improved LFS
Patkar et al., 2018 [68]	83 Yes/-	1-log cut-off between post induction and post consolidation	post induction and post consolidation	NGS-MRD for *NPM1* mutations as the most independent prognostic factor	No	NA	- NGS was an useful test for prediction of relapse and survival - NGS and MFC may be complementary, with patients MRD-negative by both techniques having excellent outcome
Onecha et al., 2018 [69]	50 Yes/7 alloHSCT	0.1% post induction/0.025% post consolidation	post induction and post consolidation	Higher risk of death in subjects with advanced age, MRD+ status or with *FLT3*-ITD	No	NA	- MRD+ status post induction and post consolidation associated with lower OS and shorter DFS and OS (33% vs 81%), respectively - NGS improved the capacity to predict AML outcome over MFC or RQ-PCR
Petrova et al., 2018 [70]	22 Yes/NA	NA	NA	*NPM1* mutation MRD more sensitive than *IDH1/2*-based MRD	NA	NA	- ddPCR more sensitive than NGS for *IDH1/2* MRD detection - In the absence of more sensitive markers, *IDH1/2* mutations can be used as reliable markers for MRD monitoring
Prata et al., 2018 [71]	0/0 (34 patients received upfront therapy with HMA)	NA	>3 log *NPM1* MRD reduction observed in 4/6 patients after 6 cycles, but three of them relapsed within <6 months of this reduction.	No factors, including type of HMA, *FLT3* and *IDH1/2* status, were prognostic of response or survival	NA	NA	- Overall response rate 45% (CR in 23.5% of patients) - Median OS 280 days - No difference in OS between cohorts with or without *NPM1* mutations suggesting limited therapeutic impact of HMA in *NPM1*-mutated AML
Ottone et al., 2018 [72]	177 Yes/NA	NA	NA	Evaluation of correlation with *DNMT3A* and *FLT3* mutations	NA	NA	- *NPM1*-mutated transcripts levels correlated with disease course, as a reliable MRD marker - *DNMT3A*^R882H^ levels did not reflect AML status
Gaksch et al., 2018 [73]	34 Yes/-	VAF <0.5%	After at least one consolidation cycle	In multivariate analysis including age, leukocyte count and genetic risk, residual disease positivity remained statistically significant as an adverse factor for RFS	NA	NA	- Persistence of non-*DNMT3A* mutations was significantly associated with higher risk of AML relapse and shorter RFS, with a trend for shorter OS. - Strong concordance between NGS and ddPCR for *NPM1* mutations

MRD, minimal/measurable residual disease; AML, acute myeloid leukemia; CHT, chemotherapy; HSCT, hematopoietic stem cell transplantation; NA; not available; alloHSCT, allogeneic HSCT; RFS, relapse-free survival; OS, overall survival; CR, complete remission; EFS, event-free survival; BM, bone marrow; PB, peripheral blood; NCN, normalized copy number; NGS, next-generation sequencing; RQ-PCR, real-time quantitative polymerase chain reaction; DFS, disease-free survival; ELN, European Leukemia Net; GO, gemtuzumab ozogamycin; MFC, multiparametric flow cytometry; TP, timepoint; ddPCR, digital droplet PCR; DTA (*DNMT3A*, *TET2*, *ASXL1*); VAF, variant allele frequency; LFS, leukemia-free survival, HMA, hypomethylating agents (azacitidine, decitabine, guadecitabine).
